# Computer simulation analysis of normal and abnormal development of the mammalian diaphragm

**DOI:** 10.1186/1742-4682-3-9

**Published:** 2006-02-17

**Authors:** Jason C Fisher, Lawrence Bodenstein

**Affiliations:** 1 Division of Pediatric Surgery, Morgan Stanley Children's Hospital of New York-Presbyterian and Department of Surgery, College of Physicians and Surgeons, Columbia University, 3959 Broadway, 216B, New York, NY 10032, USA; 2 Olana Technologies, Inc., 5424 Arlington Avenue, H51, Bronx, NY 10471, USA

## Abstract

**Background:**

Congenital diaphragmatic hernia (CDH) is a birth defect with significant morbidity and mortality. Knowledge of diaphragm morphogenesis and the aberrations leading to CDH is limited. Although classical embryologists described the diaphragm as arising from the septum transversum, pleuroperitoneal folds (PPF), esophageal mesentery and body wall, animal studies suggest that the PPF is the major, if not sole, contributor to the muscular diaphragm. Recently, a posterior defect in the PPF has been identified when the teratogen nitrofen is used to induce CDH in fetal rodents. We describe use of a cell-based computer modeling system (*Nudge++*™) to study diaphragm morphogenesis.

**Methods and results:**

Key diaphragmatic structures were digitized from transverse serial sections of paraffin-embedded mouse embryos at embryonic days 11.5 and 13. Structure boundaries and simulated cells were combined in the *Nudge++*™ software. Model cells were assigned putative behavioral programs, and these programs were progressively modified to produce a diaphragm consistent with the observed anatomy in rodents. Homology between our model and recent anatomical observations occurred under the following simulation conditions: (1) cell mitoses are restricted to the edge of growing tissue; (2) cells near the chest wall remain mitotically active; (3) mitotically active non-edge cells migrate toward the chest wall; and (4) movement direction depends on clonal differentiation between anterior and posterior PPF cells.

**Conclusion:**

With the PPF as the sole source of mitotic cells, an early defect in the PPF evolves into a posteromedial diaphragm defect, similar to that of the rodent nitrofen CDH model. A posterolateral defect, as occurs in human CDH, would be more readily recreated by invoking other cellular contributions. Our results suggest that recent reports of PPF-dominated diaphragm morphogenesis in the rodent may not be strictly applicable to man. The ability to recreate a CDH defect using a combination of experimental data and testable hypotheses gives impetus to simulation modeling as an adjunct to experimental analysis of diaphragm morphogenesis.

## Background

**Figure 1:**
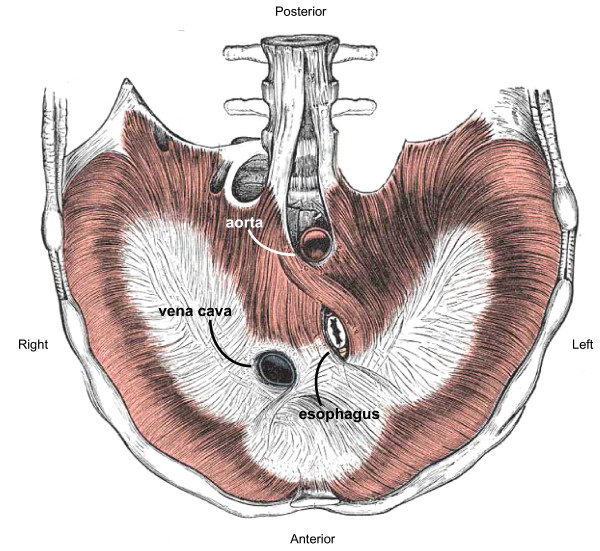
**Human diaphragm anatomy**. Drawing of a normal human diaphragm in transverse section, viewed from below (i.e., from within the abdominal cavity); after Gray [51]. Anterior-posterior orientation of all diaphragm images within this report follows the same layout that is depicted here; left-right orientation is also maintained except for Figure 2, which is viewed from the chest cavity (i.e. viewed from above) and hence is left-right reversed.

**Figure 2:**
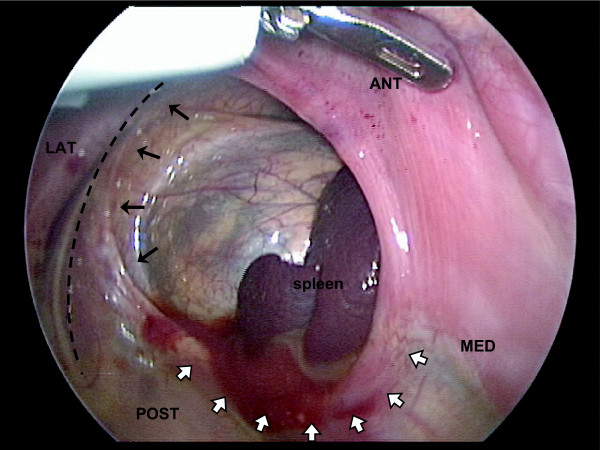
**Human CDH**. View of a human CDH during thoracoscopic surgical repair. The image is obtained through the scope, from the chest (i.e. above) and with the infant rotated on the operating table. Hence, the image is slightly rotated and left-right reversed with respect to other figures within this report. The retroperitoneum and spleen are visualized through the defect. Note that (i) the diaphragm anteriorly is intact, (ii) the defect extends to the body (chest) wall (dashed line) in the posterolateral position (solid arrows), and (iii) in the posteromedial position, a rim of diaphragm is present (open arrows). Thus surgical closure of the defect involves apposing diaphragm to chest wall laterally but diaphragm to diaphragm medially [22]. Larger defects may not be amenable to primary closure and generally are repaired with a prosthetic patch. Anterior (ANT), posterior (POST), medial (MED), and lateral (LAT). (Image courtesy of Dr. Edmund Yang, Vanderbilt Children's Hospital, Nashville, TN, USA.)

Among anomalies of human diaphragm development, Bochdalek-type posterolateral congenital diaphragmatic hernia (CDH) is of most consequence. Even as an isolated finding, CDH remains a clinical challenge with significant morbidity and mortality [1]. Despite this, developmental biologists have paid scant attention to the diaphragm as an object of study. The diaphragm is not externally visible and is devoid of the detailed morphological patterning useful in evaluating the results of experimental manipulation. Yet the gross structure of the diaphragm (essentially a curved sheet) is favorable to both experimental study and computer simulation analysis (Figs. 1, 2). Here we describe use of computer simulation to model morphogenesis of the mammalian (mouse) diaphragm. In particular, we apply a new modeling paradigm that combines experimental data and theoretical modeling in a single composite – the "Roger Rabbit" method (see footnote 1).

**Figure 3:**
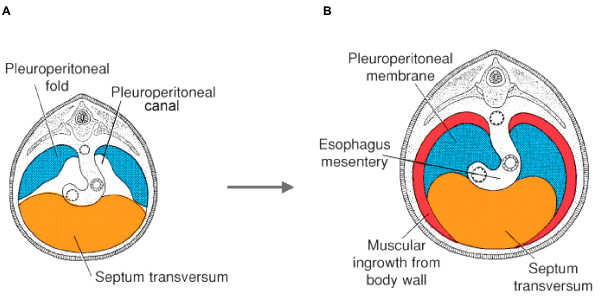
**Diaphragm morphogenesis**. Classical description of the origins of the human muscular diaphragm, depicted at 5 weeks **(A) **and 4 months **(B) **of gestation. The diaphragm is described as arising from the septum transversum, pleuroperitoneal folds (PPF), esophageal mesentery and thoracic body wall. (After Sadler [6]).

The original concepts of diaphragm development were derived from studies in descriptive embryology [2,3]. The diaphragm musculature was thought to arise as a composite from several sources: the septum transversum, the pleuroperitoneal folds (PPF), the dorsal (or esophageal) mesentery, and the thoracic body wall (Fig. 3) [4-6]. Recent studies in the rat have been invoked to challenge this view [7-10]. According to these authors, the PPF represent the overwhelmingly major, if not sole, contributors to the muscular portion of the diaphragm. Whether this difference reflects an improved understanding of diaphragm development or simply inter-species variation is not known (see Discussion).

**Figure 4:**
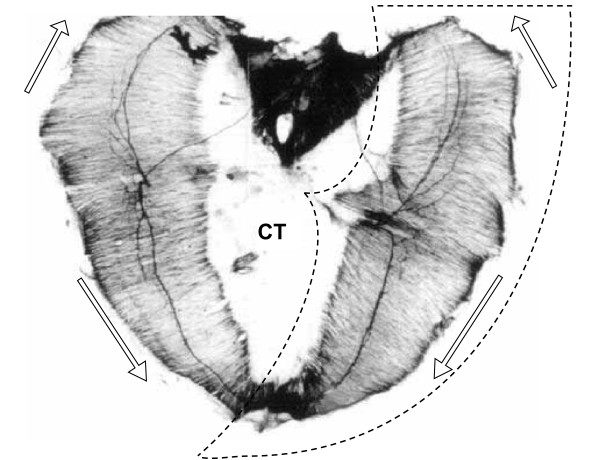
**Rodent diaphragm anatomy**. Normal E17.5 rat diaphragm whole mount, with key morphological components highlighted: curvilinear gross morphology of each muscular hemi-diaphragm (dashed line), non-muscularized central tendon (CT), anterior and posterior muscular extension along lateral body wall (hollow arrows). (Photomicrograph adapted with editorial permission from [17].)

**Figure 5:**
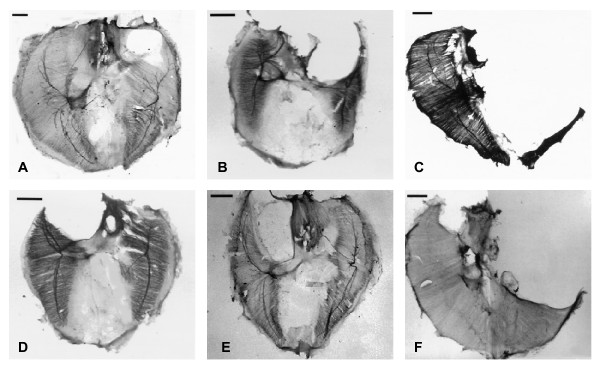
**Rodent nitrofen CDH model**. A series of nitrofen induced rat diaphragmatic hernias demonstrating the large size variation. Both left (**A–C**), right (**D**, **E**) and bilateral (**F**) hernias are figured although only left-sided defects are modeled in this report. (Reproduced and adapted with editorial permission from [10].)

**Figure 6:**
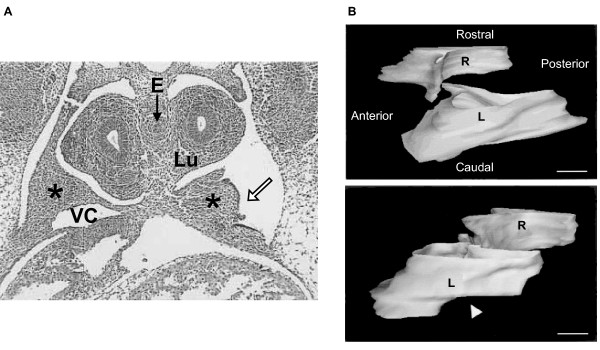
**PPF defect as a precursor to CDH in the rodent nitrofen model**. **(A) **E13.5 transverse section of embryonic rat exposed to nitrofen, with a posterior defect (hollow arrow) in the left PPF (star). The section is through the mid-portion of the PPF. Lu, lung; VC, vena cava; E, esophagus. **(B) **Reconstruction is used to define the PPF defect in three dimensions. The upper image is of normal left and right PPFs with the perspective of looking through the left lateral cervical wall of an E13.5 rat. The lower image shows a left-sided defect in the PPF (hollow arrowhead). Scale bars = 100 μm. (Images reproduced with editorial permission from [11].)

A variety of scenarios have been proposed to explain the origin of the defect in CDH. These include CDH as a consequence of abnormal lung development, CDH as a consequence of abnormal phrenic nerve innervation, CDH as a consequence of abnormal myotube formation, and CDH as a failure of closure of the embryonic pleuroperitoneal canal [9,11,12]. In the most widely-studied experimental model of CDH [13-15], pregnant rats or mice treated with the herbicide nitrofen (2,4-dichloro-phenyl-p-nitrophenyl ether) yield offspring with characteristic diaphragmatic hernias. As in the human anomaly, these experimental defects are of quite variable size (Figs. 4, 5). Examination of mid-gestation embryos in this model has revealed a defect in the posterior PPF (Fig. 6) [10]. Although the relationship of the nitrofen-induced CDH model in the rodent to the naturally-occurring human anomaly is unknown, this PPF defect is highly suggestive of a specific precursor lesion.

Here we focus on the rodent diaphragm. We investigate normal development and the abnormal development seen in the nitrofen model. We specifically examine mechanisms by which the recently-documented PPF defect in the early embryo [10] may evolve into the larger CDH defect of the later embryo and adult.

## Methods

### Histological preparation

Transverse sections of mouse embryos at stages that bracket major morphogenetic events of diaphragm development (embryonic day 11.5 and day 13 [E11.5 and E13]) have been examined (see footnote 2). Paraffin embedded mouse embryos were prepared in accordance with the standards of the Institutional Animal Care and Use Committee of Columbia University. Five micron transverse serial sections were cut and stained with hematoxylin and eosin.

### Image analysis and digitalization

**Figure 7:**
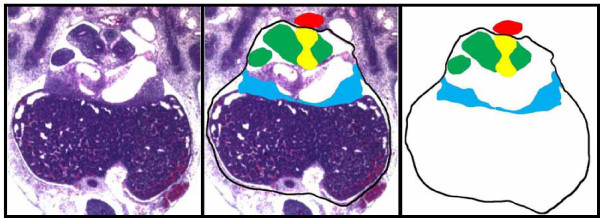
**Collection of tissue boundary data**. Transverse section of an E11.5 mouse embryo, with superimposed digital tracings of the body wall (black line), PPF (blue shading), lungs (green shading), esophageal mesentery (yellow shading) and dorsal aorta (red shading).

**Figure 8:**
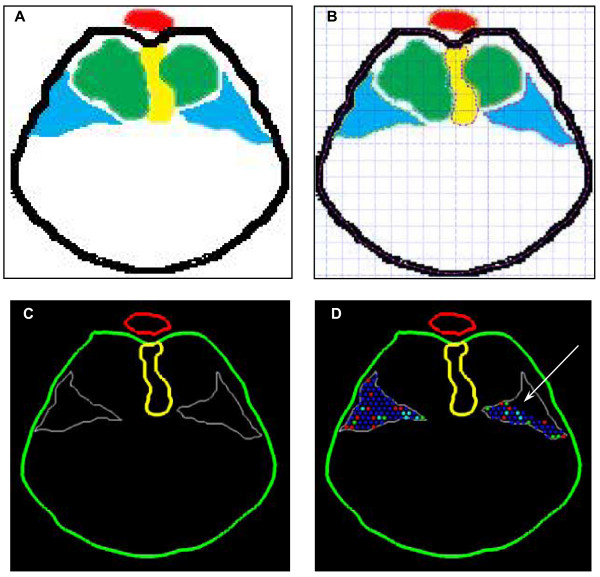
**Input of experimental images into modeling software**. **(A) **Computer-assisted tracing of anatomical boundaries relevant to diaphragm development in an E11.5 mouse (black – body wall, red – aorta, yellow – esophageal mesentery, blue – PPF, green – lungs). Where the relevant structures are not captured on a single section, these boundaries represent a composite orthographic projection of serial sections (see Fig. 5 and text). **(B) **Digital capture of coordinate-space data points along anatomical boundaries using *GetData*^© ^*2.17 *software. **(C) **Regeneration of digitized anatomical boundaries by the Nudge++™ modeling software (green – body wall, red – aorta, yellow – esophageal mesentery, white – PPF). **(D) **Nudge++ image with the PPF populated by model cells. Cells are not added to the posterolateral aspect of the left posterior PPF defect (arrow) to recreate experimental findings in the nitrofen model (see text and Fig. 6).

Sections were examined under bright microscopy at 40× magnification. Selected microscopy images were digitally captured, and computer-assisted tracing of key diaphragmatic structures was performed (Fig. 7). Where necessary, images from sequential sections were "stacked" to complete structure outlines – in essence, creating a two-dimensional orthographic projection of structures where the complete structure could not be captured on a single transverse section. Tracings were then imported into image-analysis software (*GetData*^© ^*2.17*, ) and digitized to yield two-dimensional coordinate-space data points. These digital coordinates were imported into the *Nudge++*™ software environment; the software then regenerated the original tracings as computerized anatomical boundaries within the simulations (Fig. 8).

### Computer simulations

*Nudge++*™ is a robust computer modeling system designed to study the morphogenesis of multi-cellular organisms (see footnote 3). Details of the model have been presented elsewhere [16]. In brief, model cells carry out programmed behaviors based on internal states and external cues. The model successively iterates over the cell population – evaluating cellular conditions and generating cellular activities. Tissues and organs are built from cohorts of these interacting cells. The model can be tailored to a variety of systems (both two- and three-dimensional) and includes an extensive and expandable set of cellular states and environmental cues (Table 1). The model also allows for the description of regions based on anatomical data; regional boundaries can act as constraints to cell movement.

**Table 1:** Cellular calculus within Nudge++™

***Internal States***	+	***External Cues***	****→	***Cell Actions***
age		boundaries		growth
cell-cycle phase		local position		division
phase-age		global position		movement
generation				death
clone				
lineage				

Individual model cells evaluate internal states and environmental cues and carry out specific actions based on this evaluation. Additional states, cues and actions  other than those listed can be added to each column. A cohort of model cells forms a tissue or organ. Simulations represent the pooled behavior of these cell cohorts over simulated tissue time. Details of these procedures have been presented elsewhere[16].

Here, we use *Nudge++*™ in two-dimensional mode whereby the model tissue is confined to a plane but individual cells are three-dimensional. Cells are modeled as inelastic spheres. Cell cycle time is normally distributed about a set mean (see footnote 4). When a cell divides, two daughter cells are produced, each of volume equal to one-half of that of the original cell. The orientations of cell divisions have been kept random within the plane of the diaphragm. There is no cell death. Active cell movement is used in some simulations (see below). Details of how these model cells interact on a geometric basis have been previously described [16].

Each simulation has been run a minimum of five times and representative runs are figured.

### Incorporation of data into simulations

**Figure 9:**
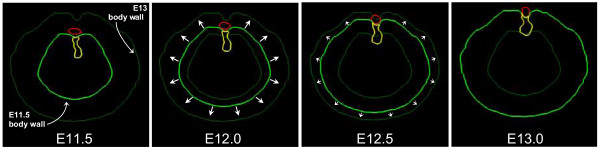
**Morphing of anatomical region boundary data over simulation time**. Shown are four images of cross-sectional tissue outlines in the model embryo and representing a transition from E11.5 to E13. Images are shown at 12 hour intervals (E11.5, E12, E12.5, and E13). Tissues outlined include the body wall (green), dorsal mesentery (yellow), PPF (white) and aorta (red). The tissue outlines in the E11.5 and E13 images are directly digitized from experimental material (see Fig. 7 and 8). The intermediate images are calculated by morphing between these two endpoints; short arrows indicate direction of body wall growth. Although only two intermediate images are shown, the program calculates new tissue outlines continuously as the simulation progresses. Those for the body wall and dorsal mesentery act as absolute boundaries to cell movement; the body wall has trophic and tropic effects in some simulations (see text).

Digitized tissue boundaries for the E11.5 and E13 mouse embryos were introduced into the simulation model as described above. Intermediate time-points for these boundaries were generated in *Nudge++*™ by a simple morphing of matching structures over embryonic time (Fig. 9).

**Figure 10:**
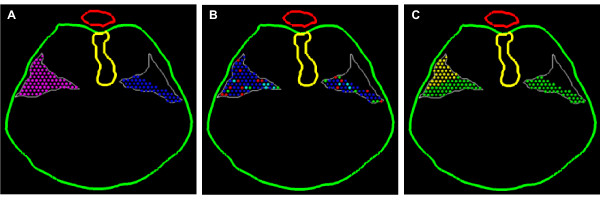
**Initial conditions**. *Nudge++*™ images of the initial PPF cell population, based on data from transverse sections of the E11.5 mouse embryo. The right side of each image models normal development; the left side models the precursor defect in the PPF and CDH development (see text and Fig. 4). The color scheme is determined by which cellular state the user chooses to observe. Pictured here is the same E11.5 simulation starting point with cells color-tagged based on **(A) **PPF of origin (purple = right, blue = left), **(B) **cell-cycle phase (blue = G1, green = S, turquoise = G2, red = M), and **(C) **polyclone (green = anterior PPF, yellow = posterior PPF).

Model cells were introduced into the initial composite based on the digitized boundaries of the PPF at stage E11.5. In each simulation, the right side is representative of a normal PPF and hemi-diaphragm, while the left side is representative of a CDH. The PPF defect has been described and defined in recent observations in the rat nitrofen CDH model [10]. Transverse sections through the mid-portion of the defective PPF demonstrate a posterolateral defect (Fig. 6). Therefore, at E11.5 the right model PPF is completely filled with cells while the cellular component of the left model PPF has a posterolateral defect (Fig. 10).

As model cells carry out program-directed behaviors within the simulation, they are physically constrained by boundaries representing the body wall and dorsal mesentery. Hence, experimentally-derived boundary data are used both to place the original model cells within the normal and defective PPF, and to modify cell behaviors over simulation time. The initial alignment of boundaries and cells is uniquely determined by the E11.5 data. However, there are options in terms of aligning the data-derived boundaries (which change over time by morphing) and the simulated cell populations (which change over time by growth, division and movement). Here we allow the coordinate space of the cell populations to "stretch" as the body wall grows (see footnote 5).

## Results

We present a series of simulations in which cellular programs are progressively modified to improve morphological fit with experimental findings in the rodent (Figs. 4, 5). We seek to match the following: (i) development of the entire muscular diaphragm from the PPF alone [8]; (ii) anterior extension of the muscular diaphragm along the chest wall, producing the image of curvilinear hemi-diaphragms and leaving a non-muscular central tendon; (iii) differentiation within each hemi-diaphragm of more central cells before more peripheral cells [17]; (iv) development of the posterior PPF defect into a larger CDH defect; and (v) normal development of the ipsilateral anterior diaphragm in CDH (isolated posterior defect). Table 2 summarizes the stepwise inclusion of these key morphological elements as they correlate with the progression of each successive simulation.

**Table 2:** Sequential achievement of modeling goals

	**Simulation I**	**Simulation II**	**Simulation III**	**Simulation IV**	**Simulation V**
	*Homogeneous Growth*	*Edge Growth*	*Edge Growth with Chest Wall Trophism*	*Edge Growth with Chest Wall Trophism and Tropism*	*Edge Growth with Chest Wall Trophism and Differential Tropism*

Development from PPF alone	+	+	+	+	+

Non-muscular central tendon	+	+	+	+	+

Central before peripheral differentiation		+	+	+	+

Anterior extension along body wall			+/-	+	+

Curvilinear morphology of hemidiaphragm				+	+

Normal anterior diaphragm ipsilateral to CDH				+/-	+

Posterior PPF persists as large CDH					+

The success of individual simulations in reproducing major observed morphological components of the developing normal and CDH diaphragm are tabulated; (+), component present; (+/-), component partially present.

### Simulation I (homogeneous growth)

**Figure 11:**
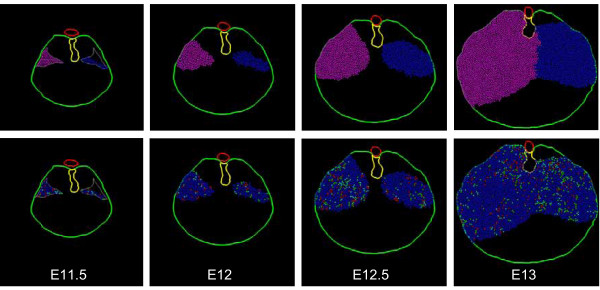
**Simulation I – homogeneous growth**. A representative simulation of the homogeneous growth pattern (see text). Tissue time runs from E11.5 to E13 (36 hours). Cell cycle time linearly increases from 6.5 h to 7.5 h over the course of the simulation. Images are taken at 12 hour intervals (E11.5, E12, E12.5, and E13). Cell color-coding is by PPF of origin (*top row*) or cell cycle phase (*bottom row*). Cell color coding conventions are as described in Fig. 8. Note the lack of cells in the anterior central position which corresponds to the non-muscular central tendon. Note also that the model hemi-diaphragms fail to adopt their characteristic curvilinear shape and the posterior PPF defect fails to enlarge significantly.

Model cells are assigned a homogeneous growth pattern in which all cells are mitotically active, all cells have the same mean cell cycle time, and all cells divide with random orientation within the plane of the simulation. There is no cell death. The experimentally derived boundaries of the body wall and dorsal mesentery act as absolute barriers to cell movement (Fig. 11). Note that the two initial cell populations expand to fill the posterior body cavity but leave an anterior-medial cell free zone that corresponds to the central tendon of the diaphragm. On the left (CDH) side, the resulting muscular diaphragm is "hypoplastic" but the initial defect in the PPF fails to propagate to generate the larger CDH defect. Also note that the enlarging left and right "polyclones" produce a fairly discrete boundary in the midline although no midline constraint is operative.

### Simulation II (edge-growth)

**Figure 12:**
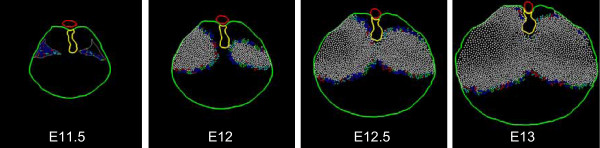
**Simulation II – edge growth**. A representative simulation of the edge growth pattern (see text). Cell cycle time, simulation length and cell color-coding conventions are as described in Figs. 10 and 11. Cell color-coding is by cell cycle phase. Note the enlarging central area of post-mitotic cells within each hemi-diaphragm (white), representing differentiation of central cells before peripheral cells. As in Simulation I (Fig. 11), the non-muscular central tendon is preserved, the left posterior PPF defect does not enlarge, and the classic hemi-diaphragm curvilinear morphology is not achieved.

Initially, this simulation follows Simulation I (normal right PPF, defect in left PPF, homogeneous growth pattern). However, beginning at mid-stage E11.5 (6 hours of simulated time), mitoses are restricted to the very edge of the tissue ('edge' refers to free-edge rather than simply edge of the PPF cell mass – cells that abut the body wall or esophageal mesentery are not considered edge cells). As expected, this generates an enlarging central area of post-mitotic cells within each hemi-diaphragm (Fig. 12). This is consistent with the findings that for each hemi-diaphragm, the more central myoblasts are the earliest to differentiate [8,17] (see footnote 6). As in Simulation I, neither the broad silhouette of the developing muscular diaphragm nor the CDH defect is well matched.

### Simulation III (edge-growth with chest-wall trophism)

**Figure 13:**
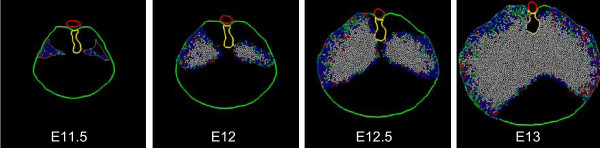
**Simulation III – edge-growth with chest-wall trophism**. Edge cells remain mitotically active; non-edge cells exit the cell cycle as their distance from the body wall increases (chest-wall trophism). Anterior diaphragm extension is enhanced over Simulation II (Fig. 12), but remains insufficient. The curvilinear shape of the hemi-diaphragm is still not achieved, the CDH defect is not enlarged, and the anterior ipsilateral diaphragm is also affected.

This simulation follows Simulation II in that edge-cells remain mitotically active. However, non-edge cells become post-mitotic with a frequency that increases with increasing distance from the body wall. This may be viewed as a trophic effect of the body wall (i.e. cells in proximity are maintained as mitotically active). The curvilinear gross morphology of each hemi-diaphragm is still lacking (Fig. 13). Only partial anterior extension of the muscular diaphragm along the body wall is present, and the CDH defect does not enlarge appropriately.

### Simulation IV (edge-growth with chest-wall trophism and tropism)

**Figure 14:**
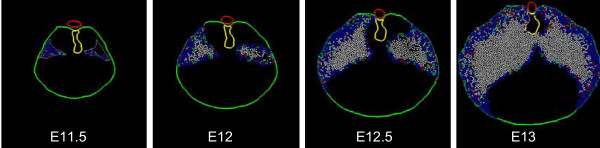
**Simulation IV – edge-growth with chest-wall trophism and tropism**. Mitotically-active non-edge cells which fall under the "trophic" effect of the body wall now actively migrate towards the body wall (tropism). Each hemi-diaphragm now adopts a more curvilinear shape. There is a more robust anterior extension of the muscular diaphragm, but the CDH defect fails to enlarge appropriately, and diaphragm tissue anterior to the CDH is somewhat hypoplastic.

This simulation follows Simulation III, except that mitotically active non-edge cells (those under the trophic influence of the chest wall) also migrate toward the body wall. In essence, the body wall both maintains these cells as mitotically active and attracts them (trophic and tropic effects). The curvilinear shape of the hemi-diaphragms is now appreciated and there is more definitive anterior extension of the muscular diaphragm along the chest wall (Fig. 14). This extension is not specifically programmed, but occurs as a consequence of cells actively moving radially (toward the body wall) and being passively displaced circumferentially (around the body wall). On the CDH (left) side, the defect does not enlarge over time, but there is some improvement in the anterior extension of the diaphragm.

### Simulation V (edge-growth with chest-wall trophism and differential tropism)

**Figure 15:**
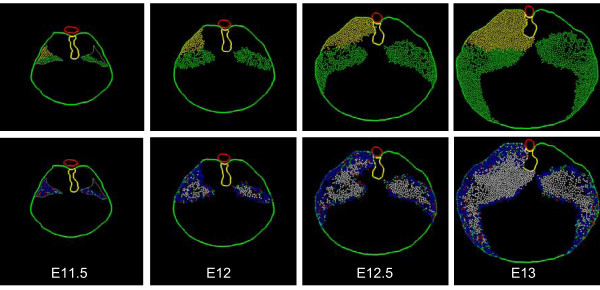
**Simulation V – edge-growth with chest-wall trophism and differential tropism**. At the start of Simulation V, cells within each PPF are designated as belonging to an anterior (green) or posterior (yellow) polyclone. The simulation series shown here are color-coded by polyclone (*top row*) or cell cycle phase (*bottom row*). Model cells display behavior as in Simulation IV (Fig. 14); however, tropism of anterior polyclone cells favors the anterior body wall, and tropism of posterior polyclone cells favors the posterior body wall (differential tropism). The diaphragm morphology, with enlarged CDH defect and more normal ipsilateral anterior diaphragm, is a better match to experimental findings (see Fig. 5).

This simulation follows Simulation IV, except that cell movement is modified on a clonal basis. Cells in the original PPF are designated as belonging to either an anterior or a posterior polyclone. These cells then migrate toward the body wall (as in Simulation IV) but anterior-derived cells add a movement component (or bias) toward the anterior body wall, and posterior cells add a similar component toward the posterior body wall (Fig. 15). For the CDH (left) side, the defect in the original PPF corresponds to the posterior polyclone and therefore no posterior-biased cells are present on this side. Note that the normal (right) side maintains the correct morphology. The CDH (left) side is now improved as a match to experimental material. First, there is propagation (enlargement) of the defect. Second, the anterior diaphragm exhibits more normal anterior extension.

## Discussion

Little is known about the growth mechanics of the developing mammalian diaphragm or the abnormalities that result in congenital diaphragmatic hernia. In particular, tissue and cell morphometrics and parameters of mitotic activity will be required to understand diaphragm morphogenesis. Treating pregnant rats and mice with the herbicide nitrofen can produce a posterior diaphragmatic defect reminiscent of that seen in human cases of CDH [13]. To what extent this rodent model is germane to the human clinical anomaly is unknown. Recent analysis of the embryonic diaphragm in the nitrofen model has defined a posterior defect in the PPF that seems to be a natural antecedent for development of the adult defect (Fig. 6) [8,10].

Our goal here has been two-fold. First, we introduce computer simulation modeling as a means for studying normal and abnormal development of the diaphragm. In doing so, we apply a novel method combining experimental data and simulated objects – the "Roger Rabbit" method. Second, we investigate specific patterns of mitotic activity and active (short-range) cell migration in simulations of normal and altered development in the nitrofen CDH model.

### Logic of simulations

We have sought to combine morphological data with simple postulates to model both normal development and the altered development of CDH. We have built our model in a stepwise fashion so that the effect of individual changes can be appreciated (Table 2). We have also limited our postulates to simple and reasonable mechanisms that are applied broadly to large, homogeneous cell populations, i.e. simple cell programs.

We begin with a homogeneous pattern of growth as a simulation "ground state." This pattern does not accurately reproduce either normal development or growth of the nitrofen-induced embryonic PPF defect into the large posterior defect of the older embryo and adult (Simulation I – Fig. 11). Evidence suggests that the mid-portion of each side of the evolving muscular diaphragm differentiates before those portions nearer the edges [8,17]. Comparable edge-based or edge-biased growth is an established pattern of mitotic activity in vertebrate embryogenesis [18,19]. We therefore institute an edge-growth pattern in which centrally located cells become post-mitotic (Simulation II – Fig. 12). In our model, this fails to generate the degree of circumferential extension noted *in vivo*. Adding a trophic effect of the body wall, whereby cells in proximity to the body wall tend to remain mitotically active, is a partial improvement (Simulation III – Fig. 13). If mitotically active non-edge cells (in essence, those cells affected by the body wall trophism) migrate toward the body wall as well, a greater degree of extension is produced (Simulation IV – Fig. 14). Similar patterns of "convergence-extension" are found extensively in early embryonic morphogenesis [20]. Here, addition of this process generates a respectable normal diaphragm, but fails to reproduce the experimental CDH finding of a large posterior defect with a normal ipsilateral anterior diaphragm. The latter can be achieved if we postulate two different cell populations within the PPF, each with a slightly different (and clonally-derived) movement pattern (Simulation V – Fig. 15). Indeed, our attempts to achieve this anterior-posterior dichotomy without some intrinsic difference in the action of anterior and posterior progenitors have not been successful. Within the context of this simulation strategy, the combination of an enlarging posterior defect and a normal anterior diaphragm does not appear possible if anterior and posterior PPF progenitors are not either (1) intrinsically distinct populations, and/or (2) responding to different environmental signals.

### Propagation of a tissue defect

**Figure 16:**
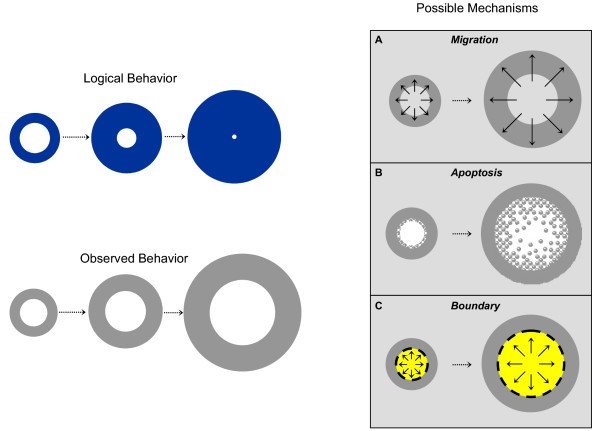
**Propagation of a tissue defect**. The images highlight the morphogenic paradox of maintaining a defect (e.g. in the embryonic PPF) when the defect is surrounded by cells that are growing and dividing. As cells divide and increase in number, they naturally tend to fill in a central defect (*logical behavior*), rather than maintain or enlarge a central defect (*observed behavior*). To explain this observed behavior (e.g. a large CDH defect from a smaller PPF defect), one must consider specific cellular actions. Possible actions that would allow the hole to actually enlarge include: (A) radial (centrifugal) cell migration; (B) position-dependent apoptosis; or (C) enlargement of a central boundary or obstacle.

Our model serves to highlight issues related to one generic component of morphogenesis – propagation of a hole or tissue defect. The defect in the early embryo PPF [8,10] seems a natural antecedent for the larger defect in the later embryo and adult. But defects do not grow of themselves; they represent the absence of surrounding tissue. As the surrounding tissue grows, the effect is to lessen and eliminate, rather than propagate, the defect. As an example, one can consider a torus (donut) of cells. As these cells divide, the natural result will be a closing of the central hole, eventually yielding a disc rather than a larger donut. To produce a larger donut (with a correspondingly larger hole) requires specific cellular interactions (Fig 16). Possible interactions include (i) active radial (centrifugal) cell migration, (ii) position-dependent cell death, and (iii) enlargement of an obstacle or boundary that forms or delineates the hole. In the simulations presented here, active migration is used. Programmed cell death has not been reported as a significant feature of diaphragm development. It has been suggested that in the rat nitrofen model, fetal liver growth within the evolving defect may contribute to enlarging the defect (the obstacle option) [21], but liver is found only occasionally in human CDH defects.

### Of mice and men

The classic description of the location of the defect in human CDH is postero-*lateral *(Fig. 1) [1,4]. There is large variation in the size and extent of the defect and large defects may extend beyond the posterolateral region and appear to involve the entire posterior aspect of the hemi-thorax. However, typically there is a posteromedial rim of diaphragm (large in the case of small defects and small to grossly non-existent in the case of large defects). This rim of posterior diaphragm is most prominent medially and fades away laterally such that the defect itself abuts the posterolateral chest wall (Fig. 2). This can be seen most clearly in moderate size defects. Very large defects may appear to have almost no posteromedial rim and thus simply seem posterior (see footnote 7); and very small defects also occur, in which the defect is completely surrounded by diaphragm without abutting the chest wall [22].

There is also considerable variation in the size of the defect in the rodent nitrofen model and, as in the human, large defects may extend across the entire posterior hemi-thorax as well as anteriorly (Fig. 5) [21]. However, the nitrofen-induced rodent defect has been described as postero-*medial *[21] (see footnote 8). This view is not without dissent. Indeed, many experienced investigators have described teratogen-induced defects [10,23], and similar defects generated by genetic [24] or nutritional [25] manipulation, as posterolateral (or, equivalently, as "dorsolateral").

**Figure 17:**
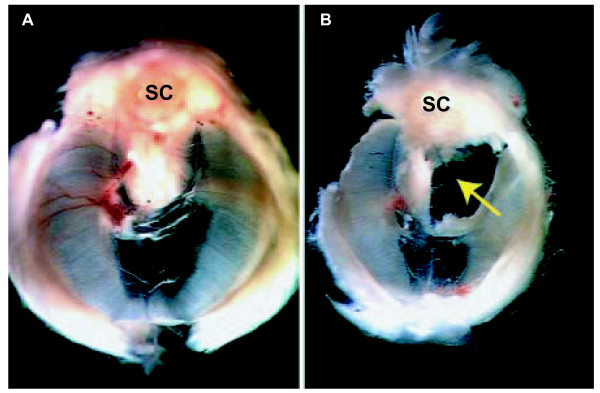
**Genetically-induced mouse CDH**. **(A) **Diaphragm from a normal (control) newborn mouse. **(B) **Left-sided diaphragmatic defect (arrow) in a COUP-TFII null mutant. Spinal cord is identified for orientation (SC). (Reproduced with editorial permission from [24]). This type of experimental defect is described as "dorsolateral" although it appears to differ in location from the classic posterolateral defect of human CDH (see text and Fig. 2).

The distinction between "posterolateral" and "posteromedial" is more than semantic to the extent that it reveals something of the embryology. Here, posterolateral is understood to describe human-type defects that generally abut the posterolateral body wall and that have a persistent posteromedial rim of diaphragm. The "morphogenic plan" that, when defective, yields such a posterolateral defect must include formation of the posteromedial rim with some degree of independence. This is not required in a plan that, when defective, yields a posteromedial defect (i.e. no posteromedial rim). Identification of this distinction should not be taken as neglect of the very real size variation that creates visual overlap at large sizes (after all, a very large medial defect will encroach laterally and a very large lateral defect will encroach medially). Published figures of rodent-type defects (Figs. 5, 17) generally do not fit the above description of posterolateral as defined in humans (Fig. 2). However, a detailed comparative analysis of the morphology of human and rodent-type defects currently is lacking, so the degree of overlap remains an open question.

The embryological origin of the human diaphragm is poorly understood [4]. The classic multi-component theory is based solely on descriptive studies and may or may not withstand scrutiny with current methods (Fig. 3) [4-6].

In contrast, the rodent provides an opportunity to create a rich experimental embryology of mammalian diaphragm development. There is now an evolving data set related to the embryology of the rodent diaphragm that targets both normal and various abnormal forms [7-11,23-27]. Thus we use findings in the rodent as a basis for our simulations. We also make the tacit assumption that differences between the mouse and the rat will be small and interchange results between these species.

According to recent studies, the rodent muscular diaphragm is formed almost exclusively from the PPF [8,10]. This contrasts with the above multi-component view of human diaphragm development. Earlier workers had described the PPF as likely a more important contributor to the diaphragm in some non-human mammals than in man [2]. It is not known whether experimental findings in the rodent indicate that the classic view of human diaphragm development is in error or whether an actual species difference exists.

The muscular diaphragm surrounds a non-muscular central tendon (Figs. 1, 4). If we consider observations in the rodent, then one feature of diaphragm morphogenesis is a circumferential extension of the PPF anteriorly along the lateral body wall. In order to generate this feature in our model (Figs. 14, 15), we programmed cells in close proximity to the body wall to remain mitotically active (a trophic effect) and to migrate toward the body wall (a tropic effect). However, if the anterolateral body wall does indeed contribute to the diaphragm in man, then this aspect of PPF extension may be unnecessary or more limited. Likewise, if in the human case a separate diaphragm component is derived from the dorsal mesentery and posteromedial body wall, then the observed posterior rim in human CDH may represent the remnant of this component, now isolated from the remainder of the diaphragm by the CDH defect (compare human CDH in Fig. 2 to rodent CDH in Fig. 5). Although differences between the human and rodent defects may reflect different pathways of pathogenesis, an alternative is that the same pathogenesis (e.g. the PPF defect previously described [10,11]) is superimposed on a slightly different underlying morphogenic plan. We find this possibility intriguing – it would link the distinct schemes for diaphragm development (multi-component in humans vs. PPF-dominated in rodents) with the disparate CDH findings (posterolateral defect with posterior rim in humans vs. posteromedial defect in rodents). Further analysis along these lines awaits a more detailed experimental analysis of human diaphragm development.

### Cell-based model

The study of morphogenesis and pattern formation has a rich history of computer simulation modeling. Simulated tissue may be modeled as a homogeneous field in diffusion and reaction-diffusion models [28-31]. Tissues may also be partitioned into mathematically useful, but not biologically defined, elements as in finite-element models and certain lattice and cellular automata models [32-35]. Although these approaches are mathematically powerful, it may be difficult to translate experimental findings into appropriate simulation parameters. Alternatively, a tissue may be partitioned into elements designed to represent actual biological cells. These latter models allow experimental findings to be more readily translated into simulations. For example, the experimental finding that a cell in a given location divides with a certain orientation is smoothly incorporated into a model that "understands" a physically defined cell, but would require some recasting to be inserted into a finite-element model and may not have a clear counterpart in a reaction-diffusion model. Cell-based models include those in which a rigid "checkerboard" [36,37] or less constrained polygonal [38-40] decomposition is used. These models usually lack the concept of extracellular space and may require *ad hoc *procedures to simulate cell division and intermingling of cells. The *Nudge++*™ model and its brethren [41,42] treat cells as independent entities. This addresses the experiment-to-simulation translation issue and readily incorporates a full range of cell "behaviors." Although different modeling strategies may be more-or-less useful in different settings, independent cell-based systems are very plastic and well suited for studying mammalian morphogenesis.

### Roger Rabbit

When computer modeling is used to simulate morphogenesis of a tissue or organ, we generally model the tissue in isolation from the surrounding embryo. Although this may be more-or-less valid when naturally bounded organs are modeled [19], we may miss important constraints and effects if we impose artificial boundaries or none at all. We have therefore developed the "Roger Rabbit" methodology for fusing experimental data with simulation modeling. This allows us to model certain features of the system (here, cells) in the context of other, non-modeled features (here, boundaries). In a clinical setting not related to morphogenesis, a similar strategy has been used to combine non-invasive imaging with finite-element modeling [43,44].

### Data limitations

Computer simulation modeling becomes more valuable as more data are accumulated. With limited extant data on the morphogenesis of the mammalian diaphragm, we are so limited (or rather, so unconstrained in possibilities) that it is difficult to select relevant modeling strategies and parameters. Put otherwise, certainly we can build a diaphragm *in silico*, but relevance to the diaphragm *in vivo *depends on our ability to link the two through experimental data. Current morphometric data on the developing mouse diaphragm include gross tissue outlines in normal animals and animals with diaphragm defects after a variety of experimental manipulations [24,27,45,46].

We limit our simulations to the time-slice between E11.5 and E13 in the mouse. These stages are chosen to bracket the events leading from the presumed anlagen (PPF) [8] to a morphologically defined (but still immature) diaphragm. We do not examine initial creation of the normal PPF or the defective PPF seen in the nitrofen model.

We have sought to model the muscular diaphragm because most of the data relate to this component. Recent studies have suggested that there may be an important, independent, non-muscular (mesenchymal) component [8]. Indeed, the CDH defect may be primary to this non-muscular component, with the muscular diaphragm following in a more passive role. Of course, we would prefer to model the determining cell population(s) but detailed information concerning the nature of the non-muscular component and its relationship to the muscular component currently is limited. Our simulations can be "redefined" to model this other component if further research focuses attention in that direction.

The topography, orientation and timing of mitotic activity within the developing diaphragm are generally unknown, although the more internal areas appear to differentiate before those nearer the edges [17]. We have therefore selected mundane cell division patterns: homogeneous and edge-based topography are compared, cell division orientation remains random, and cell cycle times are homogeneous throughout the tissue but increase slowly over time. We have not added cell death as a factor since apoptosis has not been described in the developing diaphragm. Although cell death has been described in the nitrofen model [47], it probably involves early myogenic precursors and may not be relevant to the time-space window of our simulations. Myogenic precursors, having migrated to the brachial plexus region from the cervical somites, then appear to migrate into the PPF [48]. Active cell migration within the developing diaphragm has not been described. We have therefore been cautious in postulating only limited, short-range movements occurring in proximity to the body wall and suggestive of the well-established process of convergence-extension [20].

Limitations imposed by the lack of experimental data beg the question of what type of data would be most useful and how simulation modeling can suggest avenues of experimental investigation. Embryogenesis is a period of profound growth. We believe that one of the shortcomings in understanding and modeling morphogenesis is an under-appreciation of the fact that patterning occurs not on a static field, but rather on a field undergoing tumultuous growth and remodeling. We have sought to address this in our simulations – the very boundaries of the simulations (the body wall and dorsal mesentery) change over time. In order to understand growth of the diaphragm, details of tissue morphometrics and the topography of mitotic activity over time must be determined; standard histological and immunohistochemical methods should suffice. The current simulations suggest an edge-type pattern of growth – experimental verification would strengthen the current model, non-verification would suggest a different set of simulations. Edge growth also is associated with a specific clonal pattern [49] that can be investigated in mammalian muscle tissue with current methods [50]. Such analysis would also be expected to shed light on the possible role of convergence-extension [20].

### Schematized model

We are acutely aware of the limitations of our highly schematized model in representing the subtle complexity of mammalian morphogenesis, and have treated these issues in some detail elsewhere [16]. One particular issue is that of modeling a three-dimensional (3D) structure in two dimensions (2D). Although *Nudge++*™ is capable of 3D modeling, we favor first trying to construct a 2D model of any system. Two-dimensional simulations generally are easier to construct from experimental data, are computationally less complex, and are easier to understand in terms of output. This does require that the biological system be amenable to this simplification. For example, the liver might be a structure for which this is not appropriate. As essentially a 2D sheet embedded in three-space, the diaphragm seems suitable for this approach. However, some caveats must be maintained. Although the mature diaphragm may be viewed as a sheet, it is multi-cellular in thickness and more than one type of cell forms the structure. As our knowledge of the system increases, it may become evident that including this multi-cellular thickness is essential for an adequate model. Also, we have derived the 2D outline of the PPF used in our model from sections of the 3D structure. Defects in the PPF in the nitrofen model have now been defined in 3D (Fig. 6) [11]. As our knowledge of diaphragm development increases, it may become necessary to incorporate this dimensionality into the model as well.

The above uncertainties notwithstanding, computer simulations can allow us to understand morphogenesis of the normal mammalian diaphragm and the events that underlie the abnormal development of CDH and other anomalies. In particular, they can act as proving grounds for various theories of development and as a means of understanding the results of the complex interactions that underlie mammalian development.

## List of abbreviations

CDH, congenital diaphragmatic hernia; PPF, pleuroperitoneal fold(s)

## Footnotes

**Footnote 1**: In the film Who Framed Roger Rabbit (copyright The Walt Disney Company and Amblin Pictures, 1988), animated characters are combined with real actors. Here we use the phrase "Roger Rabbit" to denote the merging of real data components with simulated cells in our computer model.

**Footnote 2**: Historically, both the mouse and rat have been used to study diaphragm development and nitrofen-induced CDH. Comparable embryonic stages are as follows: mouse (rat) – 11.5 (13), 12 (13.5), 12.5 (14), 13 (14.5), 13.5 (15), 14 (15.5).

**Footnote 3**: Nudge++™ is used under license from Olana Technologies, Inc., 5424 Arlington Avenue, H51, Bronx, New York, United States 10471.

**Footnote 4**: There are currently no data on cell size or density in the developing diaphragm. Also, data on cell cycle times are lacking. In these simulations, cell volume is set at 525 μm2 yielding a radius of about 5 μm. Alteration in absolute cell size should not affect the key features of the simulations since changes in cell size can be offset by changes in cell number and mitotic rate. Likewise, we have chosen cell cycle times as sufficient to fill the projected area of the developing diaphragm in the allotted time. In general, cycle time is designated as equal throughout the tissue but increases gradually over simulated embryonic time, i.e. mitotic rate slows as the embryo ages. Further details for each simulation are provided in the Results section and the appropriate figure legends.

**Footnote 5**: Alternatives include: (i) the PPF (cell mass) remains fixed to the body wall and moves laterally as the body wall expands, (ii) the PPF remains fixed to the dorsal mesentery, and (iii) the PPF remains fixed in free (absolute) space. These alternatives were examined for completeness but do not change the key simulation results as presented here (data not shown).

**Footnote 6**: Whereas there need not be a strict correlation between cells becoming post-mitotic and cells undergoing differentiation, this is a convenient shorthand in the present case. The actual topography of mitotic activity in the developing diaphragm is not known, nor is it known to what extent the degree of differentiation of these myoblasts coincides with mitotic activity or perhaps cell fusion.

**Footnote 7**: Although some large defects seem to have no posterior rim, many have a very small rim tucked into the retroperitoneum. Indeed, one component of the surgical repair of CDH is "unfurling" of this small, occult rim of diaphragm.

**Footnote 8**: It is important to note that medial is actually slightly off the true midline (where reside the esophagus, aorta, inferior vena cava and spine). This usage also is consistent with Morgagni-type anterior defects that are generally described as anteromedial although when unilateral they present slightly off the actual midline.
